# Molecular detection and antibiotyping of multi‐drug resistant *Enterococcus faecium* from healthy broiler chickens in Bangladesh

**DOI:** 10.1002/vms3.669

**Published:** 2021-11-16

**Authors:** Krishna Roy, Md. Saiful Islam, Anamika Paul, Samina Ievy, Mithun Talukder, Md. Abdus Sobur, Fatimah Muhammad Ballah, Md. Shahidur Rahman Khan, Md. Tanvir Rahman

**Affiliations:** ^1^ Department of Microbiology and Hygiene Faculty of Veterinary Science Bangladesh Agricultural University Mymensingh Bangladesh

**Keywords:** antibiotic resistance genes, *Enterococcus faecium*, MAR, MDR, public health

## Abstract

**Background:**

*Enterococcus faecium* is a ubiquitously distributed member of the intestinal microbiota of both humans and animals. Antibiotic resistant *E. faecium* are a major public health concern.

**Objectives:**

This study aimed to detect multi‐drug resistant (MDR) *E. faecium* and their antibiotic resistance genes from broiler chickens in Bangladesh.

**Methods:**

A total of 100 faecal samples of healthy broilers were screened by conventional methods and polymerase chain reaction (PCR) to detect *E. faecium* and their resistance genes. Disk diffusion test was employed to determine antibiotic profiles.

**Results:**

By PCR, among 100 samples, 45% [95% confidence interval (CI): 35.62%–54.76%] were positive for *E. faecium*. Based on antibiogram, all the *E. faecium* isolates were found resistant to ampicillin, and frequently (93.33%–55.56%) resistant to ceftriaxone, cefotaxime, streptomycin, erythromycin, and imipenem; moderate to lower (26.67%–4.44%) resistance to tetracycline, ciprofloxacin, norfloxacin, chloramphenicol, gentamicin, and vancomycin. Interestingly, 80% (95% CI: 66.18%–89.10%) *E. faecium* isolates were MDR in nature. In addition, the indices of multiple antibiotic resistance (MAR) ranged from 0.08 to 0.83. By bivariate analysis, high positive significant correlations were observed between resistance profiles of erythromycin and imipenem, ciprofloxacin and norfloxacin, erythromycin and streptomycin, ceftriaxone and cefotaxime, tetracycline and chloramphenicol, and streptomycin and imipenem. Furthermore, the prevalence of resistance genes of *E. faecium* was 58.33% (*tetA*), 33.33% (*tetB*), 35.56% (*bla_TEM_*), 60% (*CITM*), 13.33% (*aadA1*), and 12% (*SHV*).

**Conclusions:**

To the best of our knowledge, this is the first study in Bangladesh to detect MDR and MAR *E. faecium* and their associated resistance genes. The detection of MDR and MAR *E. faecium* and their corresponding resistance genes from healthy broilers is of public health concern because of their potential to enter into the food chain.

## Graphical abstract

This is the first study in Bangladesh to detect multidrug‐resistant (MDR) and multiple antibiotic‐resistant (MAR) *Enterococcus faecium* along with their corresponding resistance genes from healthy broilers. Our findings indicated that faecal materials of broilers harbour a high level of MDR and MAR *E. faecium* and multiple antibiotic resistance genes in those isolates. The results obtained from our study show severe health risks to both animals and humans.

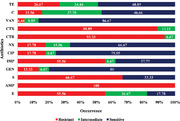

## 1 INTRODUCTION

Members of enterococci, under the family of *Enterococcaceae*, are deemed symbiotic pathogens which can develop hospital‐ and community‐acquired infections in humans and multifarious types of infections in animals (Tian et al., 2019). They are ubiquitously present in both humans’ and animals’ intestinal tracts, in addition to different environmental sources such as water and soil (Kim et al., 2019). Enterococci are used as faecal indicator organisms to track microbial sources and antibiotic resistance trends in microorganisms. Furthermore, resistance surveillance systems of humans and animals use *Enterococcus* spp. as important indicator organisms (Tyson et al., 2018).

All age groups of poultry can be affected by *Enterococcus* spp., but their devastating effects are developed in embryos and young chicks (MSD Manual Veterinary Manual, 2019). In poultry, *Enterococcus* spp. can colonize the intestine and cause disease conditions such as osteomyelitis, femoral head necrosis, spondylitis, skeletal disease, and arthritis in poultry. Additionally, *Enterococcus faecium* cause endocarditis, septicaemia, amyloid arthropathy, and spondylitis (Robbins et al., 2012). Furthermore, these organisms are directly related to musculoskeletal disease in broiler breeders and broilers (Robbins et al., 2012).

*E. faecium* along with *E. faecalis* can cause about 90% of clinical infections and more than 10% of nosocomial infections in humans (Torres et al., 2018). Importantly, *E. faecium* are deemed the fourth most dominant among human pathogens globally (Rehman et al., 2018). In humans, *Enterococcus* spp. usually develop infections in urinary and respiratory tracts, sites of surgery, skin and soft tissue, and gastrointestinal tracts (Ike, 2017). The zoonotic pathogens *E. faecium* can be transmitted from animals to humans and can develop bacteraemia, urinary tract infections, infective endocarditis, wound infections, sepsis, and meningitis (Hammerum, 2012).

Antimicrobial resistance (AMR) shows negative challenges to global public health (Rahman et al., 2020) and endangers all of the one‐health components (Islam, Nayeem, et al., 2021). Low‐ and middle‐developing countries are facing the ominous effects of AMR. AMR will cause a huge number of deaths in the world's human populace if effective and novel antimicrobial agents cannot be introduced in the future (Clifford et al., 2018). Antibiotic resistance has been promoted in poultry by the haphazard use of antibiotics in their production as growth promoters with treating bacterial infections (Talukder et al., 2021). These activities in poultry can promote bacteria to be resistant to multiple antibiotics. These resistant bacteria are usually developed in the microbiota of chickens and can easily be spread to the environment via faecal contamination (Hafez & Attia, 2020). Associating in the gut microbiota and environments (litter, surface, air, water, etc.) of poultry, these resistant bacteria can acquire resistance genes and keep persisting them for a long time after discontinuation of the antibiotic treatment (Obeng et al., 2013).

As *Enterococcus* spp. are naturally gut‐oriented pathogens, they can serve as reservoirs of resistance genes. In addition, enterococci show intrinsic resistance to multiple classes of antibiotics which assists them to acquire abilities to be highly resistance against diversified antibiotics and to be transferred horizontally to other bacteria with the help of mobile genetic determinants (Petsaris et al., 2005). Interestingly, enterococci are resistant to multiple antimicrobial drugs, for example, aminoglycosides, β–lactams, fluoroquinolones, amphenicols, macrolides, tetracyclines, and glycopeptides (Fracalanzza et al., 2007). The vast resistance characteristics of enterococci can limit therapeutic options especially antibiotic treatment in nosocomial infections in humans and in multiple kinds of diseases in poultry. Therefore, it becomes pivotal to monitor multi‐drug resistant (MDR) enterococci which have both animal and public health significance.

Globally, there are some studies that describe the detection of *E. faecium* from broiler chickens (Rehman et al., 2018; Robbins et al., 2012; Šeputienė et al., 2012), but to the best of our knowledge, there are no data available in Bangladesh that detect antibiotic resistance genes carrying *E. faecium* from broilers. In addition, the inconveniences in treating enterococci infections are connected with AMR. This study was therefore aimed to detect *E. faecium* from faecal materials of healthy broiler chickens using a molecular‐based approach along with detection of their antibiotic resistance phenotypes and genotypes.

## 2 MATERIALS AND METHODS

### 2.1 Sample size calculation

As there was no research on molecular detection of *Enterococcus* spp. in Bangladesh, the sample size was calculated with the 50% assumptive prevalence and the 95% confidence interval (CI). The previously described (Thrusfield, 1995) formula of sample size calculation was as follows: *n* = Z^2^pq/d^2^, where *n* = desired sample size, *Z* = the normal standard deviation (1.96 at 95% CI), *p* = prevalence (50% or 0.5), *q* = (1‐p) = (1–0.5) = 0.5, *d* = precision (10% or 0.1). So, *n* = (1.96)^2^ × 0.5 × 0.5/ (0.1)^2 ^= 96.04. Therefore, we collected 100 faecal samples aseptically from broiler chickens.

### 2.2 Study area and sampling

**FIGURE 1:**
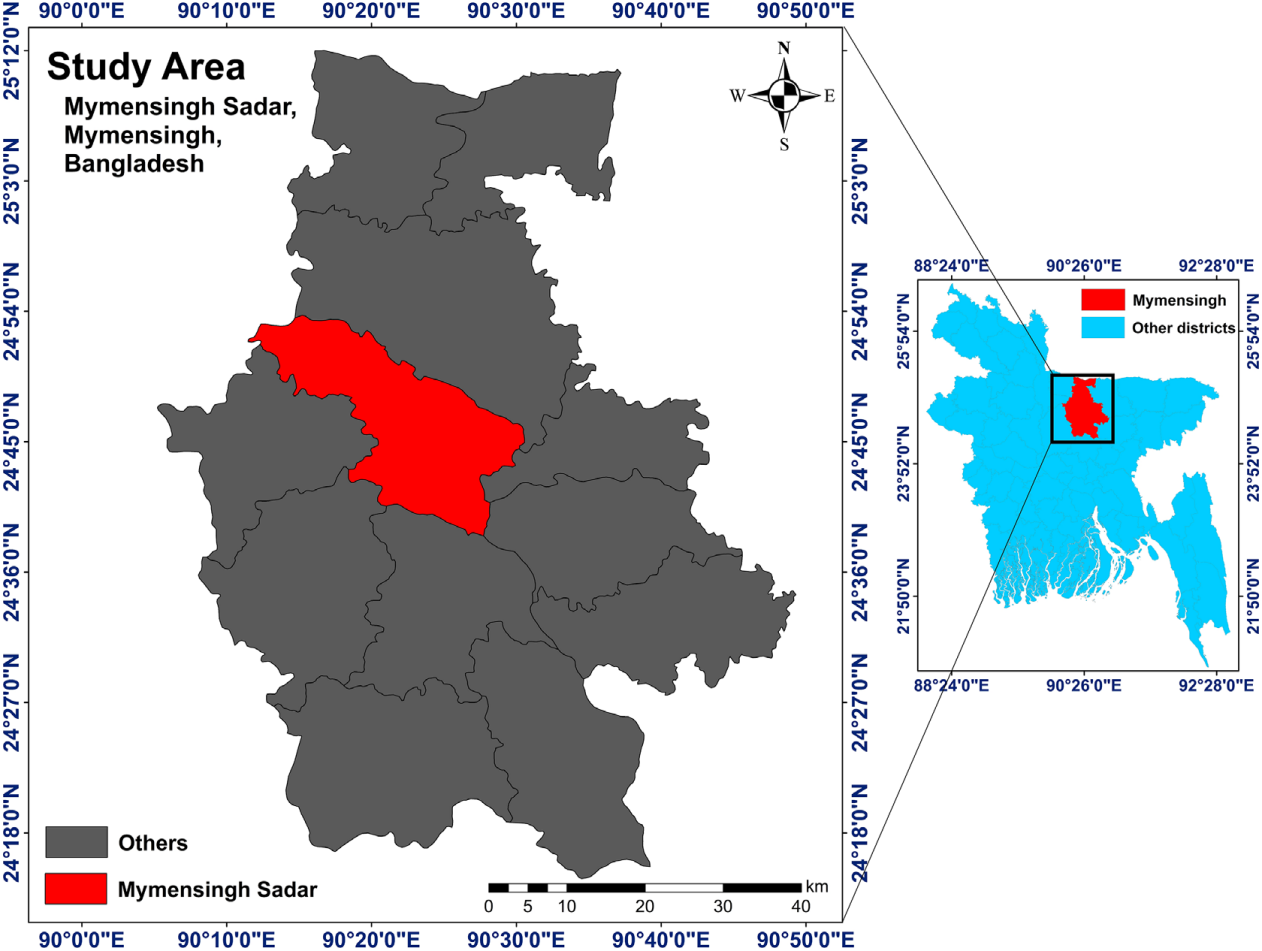
Study area map of the present study. The map was created with ArcMap 10.7 software (ESRI, Redlands, CA, USA)

From July 2019 to March 2021, this study was performed in different poultry farms within Mymensingh Sadar Upazila (24.7851° N, 90.3560° E), Mymensingh, Bangladesh. The study area is audited in Figure 1. A total of 100 freshly dropped faecal samples of broilers were collected aseptically. Each sample was collected by swirling sterile cotton buds into faecal materials and taken into a sterile zip‐lock bag having a particular tag number. After collection, samples were brought to the laboratory under cold chain maintenance and seeded immediately to 5 ml nutrient broth containing sterile test tubes. The test tubes were then incubated in aerobic condition at 37°C for 18–24 h.

### 2.3 Isolation of *Enterococcus* spp

Initial isolation of *Enterococcus* spp. was carried out by culturing on enterococcus agar base (EAB) (HiMedia, India) media. For this purpose, one loopful cultured broth was streaked on EAB media and subsequently incubated for optimum condition (aerobically at 37°C overnight). Oval‐shaped yellowish colonies on EAB media were initially presumed as *Enterococcus* spp., and further confirmed by Gram's staining and biochemical tests, for example, sugar fermentation tests, Voges–Proskauer test, indole test, and catalase test (Facklam et al., 2002).

### 2.4 Molecular detection of *E. faecium*

Isolated *Enterococcus* spp. were subjected to simplex polymerase chain reaction (PCR) to detect *E. faecium* targeting *ddl_E. faecium_* gene (Table 1).

**TABLE 1:** Primers used in the present study

Target genes	Primer Sequence (5′–3′)	Amplicon size (bp)	Annealing temperature (°C)	References
*ddl_E. faecium_*	F: GCAAGGCTTCTTAGAGA R: CATCGTGTAAGCTAACTTC	550	50	Dutka‐Malen S et al., 1995
*tetA*	F: GGTTCACTCGAACGACGTCA R: CTGTCCGACAAGTTGCATGA	577	57	Randall et al., 2004
*tetB*	F: CCTCAGCTTCTCAACGCGTG R: GCACCTTGCTGATGACTCTT	634	56	Randall et al., 2004
*CITM*	F: TGGCCAGAACTGACAGGCAAA R: TTTCTCCTGAACGTGGCTGGC	462	47	Van et al., 2008
*blaTEM*	F: CATTTCCGTGTCGCCCTTAT R: TCCATAGTTGCCTGACTCCC	793	56	Randall et al., 2004
*ereA*	F: GCCGGTGCTCATGAACTTGAG R: CGACTCTATTCGATCAGAGGC	419	52	Van et al., 2008
*SHV*	F: TCGCCTGTGTATTATCTCCC R: CGCAGATAAATCACCACAATG	768	52	Van et al., 2008
*aadA1*	F: TATCCAGCTAAGCGCGAACT R: ATTTGCCGACTACCTTGGTC	447	58	Van et al., 2008

For the PCR, the genomic DNA was extracted from isolated *Enterococcus* spp. by the boiling method as previously described (Ievy et al., 2020). In brief, initially, 1 ml previously enriched culture was centrifuged at 5000 rpm for 5 min; subsequently, the supernatant was discarded, followed by preparation of suspension by adding 200 μl phosphate buffer solution. The suspension was then boiled and cooled for 10 min in each step and centrifuged for 10 min at 10,000 rpm. Finally, the supernatant was collected as genomic DNA and stored at −20°C for further research.

All the PCR were done in a final volume of 20 μl reaction [nuclease free water 4 μl, master mixture (2×, Promega, Madison, WI, USA) 10 μl, forward and reverse primer 1 μl for each, and genomic DNA 4 μl. After completing amplification, the PCR products were visualized by running in 1.5% agarose gel electrophoresis, followed by staining in ethidium bromide, documenting under ultraviolet trans‐illuminator (Biometra, Göttingen, Germany). Note that 100 bp and 1 kb DNA ladder were employed to check the expected band size of the amplified PCR products (Promega).

### 2.5 Antibiotic susceptibility testing

Kirby–Bauer disk diffusion test (Bauer, 1966) was employed to evaluate the antibiotic susceptibility of isolated *E. faecium*. The test was done by spreading freshly growth cultures (equivalence to 0.5 McFarland solution) on Mueller–Hinton agar (HiMedia) plates. Here, nine classes of antibiotic comprising 12 antibiotics were employed: fluoroquinolones (ciprofloxacin 5 μg and norfloxacin 10 μg), glycopeptides (vancomycin 30 μg), tetracyclines (tetracycline‐ 30 μg), aminoglycosides (gentamicin 10 μg, streptomycin 10 μg), penicillins (ampicillin 25 μg), macrolides (erythromycin 15 μg), amphenicols (chloramphenicol 30 μg), carbapenems (imipenem 10 μg), and cephalosporins (ceftriaxone 30 μg and cefotaxime 30 μg). The results (resistant, intermediate, and sensitive) were interpreted by following the guidelines of the Clinical and Laboratory Standards Institute (CLSI) (CLSI, 2018), and where not possible, according to the European Committee on Antimicrobial Susceptibility Testing (EUCAST, 2019). Isolates showing resistance to three or more classes of antibiotics were recorded as MDR (Sweeney et al., 2018). Moreover, the multiple antibiotic resistance (MAR) index was calculated by the following formula: MAR = *m*/*n*, here ‘*m*’ implies the number of antibiotics resistance to a particular *E. faecium* isolate and ‘*n*’ implies the total number of antibiotics used (Krumperman, 1983).

### 2.6 Detection of antibiotic resistance genes

Simplex PCR was employed to detect resistance genes of *E. faecium* isolates associate with tetracycline (*tetA* and *tetB*), ampicillin (*bla_TEM_* and *CITM*), erythromycin (*ereA*), imipenem (*SHV*), and streptomycin (*aadA1*). The primers and targeted genes are documented in Table 1.

### 2.7 Statistical analysis

#### 2.7.1 Descriptive analysis

Data were initially brought into Excel‐2013 (Microsoft Office 2013; Microsoft, Los Angeles, CA, USA) and subsequently exported to Statistical Package for Social Science (IBM SPSS 25; IBM, Chicago, IL, USA) and Graphpad Prism version 8.4.3 (GraphPad Software, Inc.) to perform descriptive and bivariate analysis. Wilson/Brown Hybrid method (Brown et al., 2001) was followed to enumerate binomial 95% CI by Graphpad Prism.

#### 2.7.2 Bivariate analysis

Pearson correlation was performed by SPSS to observe the potential association between any of the two antibiotics that were resistant to *E. faecium* isolates. The statistically significant *p*‐value was fixed at 0.05.

## 3 RESULTS

### 3.1 Prevalence of *E. faecium*

Among 100 faecal samples of broiler chickens, 88 (88%; 95% CI: 80.19%–93.00%) were found positive for *Enterococcus* spp. based on their cultural, staining, and biochemical properties; of which 45 samples (45%, 95% CI: 35.62%–54.76%) were confirmed as *E. faecium* by *ddl* gene targeted PCR.

### 3.2 Antibiotic susceptibility testing

**FIGURE 2:**
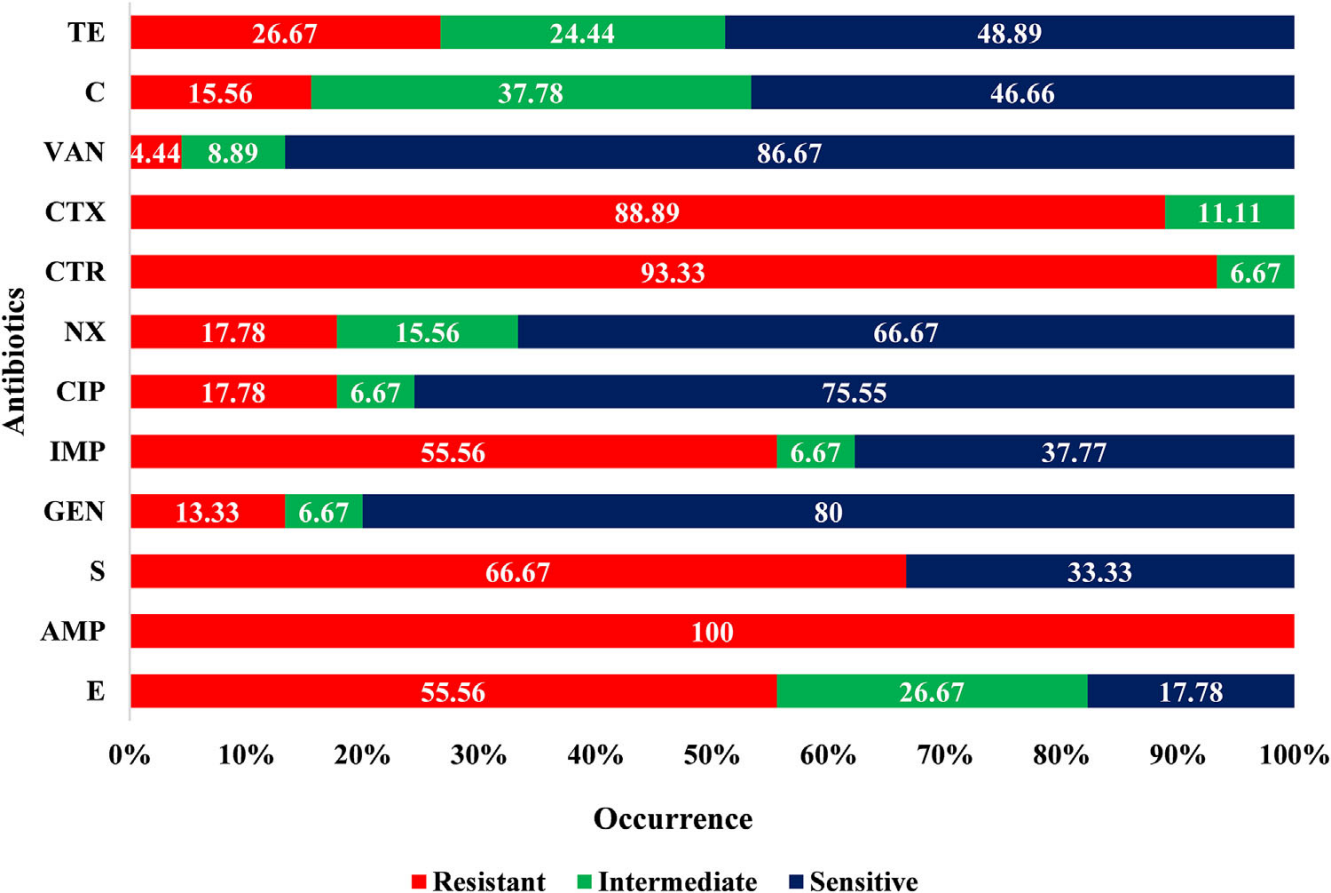
Antibiogram profiles of *Enterococcus faecium* isolated from faecal materials of healthy broiler chickens. Abbreviations: AMP, ampicillin; C, chloramphenicol; CIP, ciprofloxacin; CTR, ceftriaxone; CTX, cefotaxime; E, erythromycin; GEN, gentamicin; IMP, imipenem; NX, norfloxacin; S, streptomycin; TE, tetracycline; VAN, vancomycin

From antibiotic susceptibility test, all the *E. faecium* isolates (*n* = 45) were phenotypically resistant to ampicillin (95% CI: 92.14%–100%) and frequently resistant to ceftriaxone (93.33%; 95% CI: 82.14%–97.71%), cefotaxime (88.89%; 95% CI: 76.50%–95.16%), streptomycin (66.67%; 95% CI: 52.07%–78.64%), erythromycin (55.56%; 95% CI: 41.18%–69.06%), and imipenem (55.56%; 95% CI: 41.18%–69.06%). Moderate to lower resistance of *E. faecium* isolates were observed against tetracycline (26.67%; 95% CI: 15.97%–41.04%), ciprofloxacin (17.78%; 95% CI: 9.29%–31.33%), norfloxacin (17.78%; 95% CI: 9.29%–31.33%), chloramphenicol (15.56%; 95% CI: 7.75%–28.78%), gentamicin (13.33%; 95% CI: 6.26%–26.18%), and vancomycin (4.44%; 95% CI: 0.79%–14.83%). The overall antibiogram profiles are represented in Figure 2.

By bivariate analysis, high positive significant correlations were audited between resistance profiles of erythromycin and streptomycin (Pearson correlation coefficient, *ρ *= 0.791; *p *= < 0.001), erythromycin and imipenem (*ρ *= 0.910, *p *= < 0.001), streptomycin and imipenem (*ρ *= 0.696, *p *= < 0.001), ceftriaxone and cefotaxime (*ρ *= 0.756, *p *= 0.001), ciprofloxacin and norfloxacin (*ρ *= 0.848, *p *= < 0.001), and tetracycline and chloramphenicol (*ρ *= 0.712, *p *= < 0.001). In addition, moderate to lower positive significant correlations were observed between chloramphenicol and erythromycin (*ρ *= 0.384, *p *= 0.009), erythromycin and norfloxacin (*ρ *= 0.299, *p *= 0.046), norfloxacin and imipenem (*ρ *= 0.299, *p *= 0.046), erythromycin and chloramphenicol (*ρ *= 0.384, *p *= 0.009), chloramphenicol and streptomycin (*ρ *= 0.303, *p *= 0.043), chloramphenicol and imipenem (*ρ *= 0.384, *p *= 0.009), tetracycline and vancomycin (*ρ *= 0.358, *p* = 0.016), erythromycin and gentamicin (*ρ *= 0.351, *p *= 0.018), and gentamicin and imipenem (*ρ *= 0.351, *p *= 0.018). Furthermore, moderately negative significant correlation was seen between resistance profiles of gentamicin and ceftriaxone (*ρ *= −0.419, *p *= 0.004). The overall outcomes of bivariate analysis are given in Table 2.

**TABLE 2:** Pearson correlation coefficients for pairs of antibiotics to assess antibiotic‐resistant *Enterococcus faecium* isolates from faecal samples of healthy broiler chickens

		E	AMP	S	IMP	NX	CTR	CTX	VAN	CIP	C	TE	GEN
E	Pearson correlation	1											
	Significance (two‐tailed)	‐											
AMP	Pearson correlation	.^a^	.^a^										
	Significance (two‐tailed)	‐	‐										
S	Pearson correlation	0.791**	.^a^	1									
	Significance (two‐tailed)	.000	‐	‐									
IMP	Pearson correlation	0.910**	.^a^	0.696**	1								
	Significance (two‐tailed)	.000	‐	.000	‐								
NX	Pearson correlation	0.299*	.^a^	0.205	0.299*	1							
	Significance (two‐tailed)	.046	‐	.176	.046	‐							
CTR	Pearson correlation	−0.060	.^a^	0.000	−0.060	0.124	1						
	Significance (two‐tailed)	.697	‐	1.000	.697	.416	‐						
CTX	Pearson correlation	−0.032	.^a^	0.050	0.111	0.164	0.756**	1					
	Significance (two‐tailed)	.837	‐	.744	.469	.281	.000	‐					
VAN	Pearson correlation	−0.024	.^a^	−0.076	−0.024	−0.100	0.058	0.076	1				
	Significance (two‐tailed)	.875	‐	.619	.875	.512	.707	.619	‐				
CIP	Pearson correlation	0.182	.^a^	0.082	0.182	0.848**	0.124	0.164	−0.100	1			
	Significance (two‐tailed)	.232	‐	.591	.232	.000	.416	.281	.512	‐			
C	Pearson correlation	0.384**	.^a^	0.303*	0.384**	−0.039	−0.131	−0.043	0.205	−0.039	1		
	Significance (two‐tailed)	.009	‐	.043	.009	.798	.391	.777	.177	.798	‐		
TE	Pearson correlation	0.034	.^a^	0.000	0.034	−0.149	−0.040	0.053	0.358*	−0.018	0.712**	1	
	Significance (two‐tailed)	.826	‐	1.000	.826	.329	.793	.728	.016	.909	.000	‐	
GEN	Pearson correlation	0.351*	.^a^	0.277	0.351*	−0.011	−0.419**	−0.277	0.233	−0.011	0.192	0.059	1
	Significance (two‐tailed)	.018	‐	.065	.018	.941	.004	.065	.124	.941	.205	.700	‐

*Note*: A *p*‐value <.05 was deemed as statistically significant.

Abbreviations: AMP, ampicillin; C, chloramphenicol; CIP, ciprofloxacin; CTR, ceftriaxone; CTX, cefotaxime; E, erythromycin; GEN, gentamicin; IMP, imipenem; NX, norfloxacin; S, streptomycin; TE, tetracycline; VAN, vancomycin.

^a^ Cannot be computed because at least one of the variables is constant.

* Correlation is significant at the 0.05 level (two‐tailed).

** Correlation is significant at the 0.01 level (two‐tailed).

### 3.3 Determination of MDR and MAR profiles of *E. faecium*

Of 45 *E. faecium* isolates, 36 isolates (80%; 95% CI: 66.18%–89.10%) were phenotypically MDR in nature. A total of 18 MDR patterns were observed, of which 22.22% (8/36; 95% CI: 11.72%–38.09%) isolates exhibited the resistance pattern number 9 (E‐AMP‐S‐IMP‐CTR‐CTX). Two isolates were resistant against 10 antibiotics under eight classes (Patterns 1 and 2). The ranges of MAR indices of *E. faecium* isolates were 0.08–0.83. Interestingly, 97.78% (44/45; 95% CI: 88.43%–99.89%) of isolates showed resistance against two or more antibiotics (Table 3).

**TABLE 3:** Multi‐drug resistance and multiple antibiotic resistance profiles of *Enterococcus faecium* isolated from faecal materials of broiler chickens

Pattern Number	Antibiotic resistance patterns	Number of antibiotics (classes)	Number of isolates	Overall MDR isolates (%)	MAR index
1	E, AMP, S, IMP, CTR, CTX, VAN, C, TE, GEN	10 (8)	1	36/45 (80)	0.83
2	E, AMP, S, IMP, NX, CTR, CTX, CIP, C, TE	10 (8)	1		
3	E, AMP, S, IMP, NX, CTR, CTX, CIP, GEN	9 (6)	1		0.75
4	E, AMP, S, IMP, CTR, CTX, C, TE	8 (7)	4		0.67
5	E, AMP, S, IMP, NX, CTR, CTX, CIP	8 (6)	4		
6	E, AMP, S, IMP, CTR, CTX, GEN	7 (5)	2		0.58
7	E, AMP, S, IMP, NX, CTR, CTX	7 (6)	1		
8	E, AMP, S, IMP, C, TE, GEN	7 (6)	1		
9	E, AMP, S, IMP, CTR, CTX	6 (5)	8		0.50
10	AMP, CTR, CTX, VAN, TE	5 (4)	1		0.42
11	AMP, S, CTR, CTX, TE	5 (4)	1		
12	E, AMP, S, IMP, GEN	5 (4)	1		
13	AMP, CTR, CTX, CIP, TE	5 (4)	1		
14	AMP, NX, CTR, CTX, CIP	5 (3)	1		
15	E, AMP, S, CTR	4 (4)	1		0.33
16	AMP, CTR, CTX, TE	4 (3)	2		
17	AMP, IMP, CTR, CTX	4 (3)	1		
18	AMP, S, CTR, CTX	4 (3)	4		
19*	AMP, CTR, CTX	3 (2)	7	–	0.25
20*	AMP, CTR	2 (2)	1	–	0.17
21*	AMO	1 (1)	1	–	0.08

Abbreviations: AMP, ampicillin; C, chloramphenicol; CIP, ciprofloxacin; CTR, ceftriaxone; CTX, cefotaxime; E, erythromycin; GEN, gentamicin; IMP, imipenem; MAR, multiple antibiotic resistance; NX, norfloxacin; S, streptomycin; TE, tetracycline; VAN, vancomycin.

* Non multi‐drug resistant.

### 3.4 Prevalence of resistance genes

By PCR, resistance gene *tetA* and *tetB* were found to be positive in 58.33% (7/12; 95% CI: 31.95%–80.67%) and 33.33% (4/12; 95% CI: 13.81%–60.94%) tetracycline resistant *E. faecium* isolates respectively; resistance gene *bla_TEM_* and *CITM* were positive in 35.56% (16/45; 95% CI: 23.22%–50.16%) and 60% (27/45; 95% CI: 45.45%–72.98%) ampicillin resistant *E. faecium* isolates respectively; *aadA1* gene was found in 13.33% (4/30; 95% CI: 5.31%–29.68%) streptomycin resistant *E. faecium* isolates, and 12% (3/25; 95% CI: 4.17%–29.96%) imipenem resistant *E. faecium* isolates were positive for *SHV* resistance gene. However, all the erythromycin resistant *E. faecium* isolates were found negative for resistance *ereA* gene (Table 4).

**TABLE 4:** Prevalence of antibiotic resistance genes of *Enterococcus faecium* isolated from faecal materials of broiler chickens

Name of antibiotics	Name of resistance genes	Prevalence (%)	95% CI (%)
Tetracycline (*n* = 12)	*tetA*	7 (58.33)	31.95–80.67
	*tetB*	4 (33.33)	13.81–60.94
Erythromycin (*n* = 25)	*ereA*	0 (0)	0.00–13.32
Ampicillin (*n* = 45)	*blaTEM*	16 (35.56)	23.22–50.16
	*CITM*	27 (60)	45.45–72.98
Streptomycin (*n* = 30)	*aadA1*	4 (13.33)	5.31–29.68
Imipenem (carbapenem) (*n* = 25)	*SHV*	3 (12)	4.17–29.96

Abbreviation: CI, confidence interval.

## 4 DISCUSSION

*Enterococcus* spp. are considered opportunistic pathogens in both humans and animals. They represent challenges in infection control due to their rapid acquisition of antibiotic resistance capabilities. The dissemination of AMR in *Enterococcus* spp. associated with poultry in Bangladesh is not detailed till now. This study was therefore investigated to get better insights on the MDR and MAR observed in *E. faecium* isolates and their corresponding resistance genes from healthy broiler chickens in Bangladesh.

In the present investigation, *E. faecium* was found positive in 45% (45/100) faecal samples of broiler chickens. The high detection rate of *E. faecium* from the faecal materials of broilers is not unusual, as *E. faecium* is a ubiquitously commensal microorganism and is considered as a part of the intestinal microbiota of humans and animals (Dubin & Pamer, 2018). In addition, enterococci are present in faecal materials of different birds, mammals, reptiles, and even insects (Dubin & Pamer, 2018). Previously, Banik et al. (2018) isolated *Enterococcus* spp. from chicken in Bangladesh, but they used only conventional methods and did not carry any molecular approach, for example, PCR assay which was used in our present study. PCR is a robust, sensitive, and rapid method in detecting *Enterococcus* spp. from any kind of sample and gives higher specificity and sensitivity of the results (Maheux et al., 2011). Globally, several studies detected *E. faecium* from broiler chickens with variable occurrence rates (Garcia‐Migura et al., 2005; Karunarathna et al., 2017; Rehman et al., 2018; Tremblay et al., 2011). These variations might be lined with the variations in geographical and seasonal distributions, the farm management systems (hygiene, biosecurity, and sanitary), sample size and types, and methodological factors (Islam, Paul, et al., 2021).

The presence of *E. faecium* in faecal materials of broiler chickens reveals that the droppings of broiler chickens can shed *E. faecium* to other birds of the farm. In addition, contaminated faecal materials can act as vehicles to contaminate water and broiler feed. Furthermore, the enterococci contamination can be transferred into the production systems via the contaminated faeces, water, or broiler feeds. Broiler meat and its products can be contaminated by enterococci that can be transmitted to the food chain and pose a potential human health concern. Enterococci can grow under extreme temperature up to 72°C which indicates that consumption of undercooked poultry meat and products has the potential to transmit enterococci to humans (Martinez et al., 2003). Furthermore, this may reveal a high risk to human health by exposure to colonized birds or by the introduction of poultry meat contaminated with enterococci or by cross‐contaminating with ready‐to‐eat foods (Obeng et al., 2013).

Any microorganism resistant to the antibiotic is a threat to human health. Results of phenotypic antibiotic susceptibility test from our present study showed that a high number of *E. faecium* isolates were resistant to ampicillin, ceftriaxone, cefotaxime, streptomycin, erythromycin, imipenem, and tetracycline. High level resistance of *E. faecium* to streptomycin is not unusual. Streptomycin is under the aminoglycoside class of antibiotics which show intrinsic resistance to enterococci. Clinically achievable concentrations of aminoglycosides are unable to enter into the cell of enterococci especially *E. faecalis*, and develop enzyme‐mediated resistance in the ribosomal target site of enterococci especially *E. faecium*. These two factors enable enterococci to show intrinsic resistance against aminoglycoside class of antibiotics (Bertelloni et al., 2015). But in contrast, we found that another aminoglycoside‐gentamicin was highly sensitive or intermediately sensitive to *E. faecium* (more than 85% isolates). This variation might have a linkage with the level of concentrations of gentamicin, as enterococci show intrinsic resistance to only a low level of concentrations of aminoglycosides (Lefort et al., 2000). Previously, Tremblay et al. (2011) also found that high proportion of enterococci was sensitive to gentamicin. However, intrinsic resistance of enterococci either to streptomycin or to gentamicin has not been illustrated properly till now (Bertelloni et al., 2015).

Similarly, enterococci also are naturally resistant to cephalosporins which are lined with the higher resistance to the cephalosporin class of antibiotics – ceftriaxone and cefotaxime – obtained from our present study. Enterococci acquire intrinsic resistance to cephalosporins by a penicillin‐binding protein (Pbp5), a transduction system (CroRS), an enzyme to synthesize peptidoglycan precursors (MurAA), and a transmembrane kinase (Ser/Thr) (Kristich et al., 2014). This trait is well defined for *E. faecalis*, but for *E. faecium*, it is not well developed yet. However, gentamicin is vastly used in enterococcal infections with the combination of beta‐lactam antibiotics or glycopeptides for obtaining synergistic bactericidal effects (Emaneini et al., 2016).

Interestingly, 55.56% of *E. faecium* isolates were resistant to imipenem which reveals an alarming condition to both human and animal health facilities. Imipenem is under the carbapenem group of antibiotics which are only exercised for treating severe bacterial infections in humans (Lamb et al., 2002). *E. faecium* acquires resistance by two distinct mechanisms: one is incremented via Pbp5 which shows low‐affinity to β‐lactam antibiotics, and another one is the mutation that occurred in Pbp5 (Joste et al., 2019). Acquisition of erythromycin and tetracycline resistance in enterococci is usually developed by mobile genetic elements (Emaneini et al., 2016). *E. faecium r*esistance to tetracycline shows importance because of its relatedness with the resistance profiles of other antibiotics (Hammerum, 2012). Erythromycin is under the macrolides class of antibiotics which have been classified as ‘critically important in human medicine’ showing importance in the treatment of different bacterial infections (WHO, 2015). We have found 4.44% vancomycin‐resistant *E. faecium* in this study which is also alarming to human and animal health. Vancomycin‐resistant *E. faecium* are WHO priority 2 high category pathogens (WHO, 2017).

By bivariate analysis, high positive significant correlations were audited between resistance profiles of erythromycin and streptomycin, erythromycin and imipenem, streptomycin and imipenem, ceftriaxone and cefotaxime, ciprofloxacin and norfloxacin, and tetracycline and chloramphenicol. The high correlation between ceftriaxone and cefotaxime, and ciprofloxacin and norfloxacin is not unusual as they are under a similar class of antibiotics (first two are under cephalosporins, and second two under fluoroquinolones). The other strong correlations might be due to the haphazard use of antibiotics in broilers. The importance of these findings is linked with developing resistance in *E. faecium* against other used antimicrobials (Ievy et al., 2020).

In the present study, we detected *tetA* (58.33%), *tetB* (33.33%), *blaTEM* (35.56%), *CITM* (60%), *aadA1* (13.33%), and *SHV* (12%) genes in *E. faecium* isolates which were responsible for corresponding antibiotic resistance. No isolates were found positive for *ereA* gene. However, the presence of different resistance genes in *E. faecium* isolates might be due to the mobile genetic elements. The gene *tetA* along with *tetC* gene form one genetic group which shares approximately 78% of the amino acid sequences in common with each other. In addition, overlapping gene *tetA* encodes a classical efflux protein and a second gene *tetB* which codes for a protein that seems to be related to the tetracycline ribosomal protection proteins (Roberts, 1996). The results of *tetA* and *tetB* from tetracycline resistant *E. faecium* isolates show that the original mechanism of tetracycline resistance generated from broiler chickens is by active efflux system. Resistance gene *bla_TEM_*, *CITM*, and *SHV* are associated with β‐lactam antibiotics. These genes presenting in the organism have abilities to inactivate the antibiotics by hydrolyzing the β‐lactam ring (Livermore, 1995). The gene cassette *aadA1* is associated with the resistance to streptomycin which generally presents in class 1 integrons related to transposons *Tn21* (Rodríguez et al., 2006). Through conjugative plasmids, this resistance gene has the ability to be horizontally transferred from *E. faecium* to other bacteria (Nde & Logue, 2008). The presence of resistance genes of *E. faecium* in broiler chickens reveals public health significance as they can be transmitted to humans via the contaminated food supply chain and/or close contact with the animals. In addition, these resistance genes can assist in the emergence of MDR and MAR bacteria in both humans and animals.

Infections caused by MDR and MAR bacteria can have serious health repercussions for humans (Urmi et al., 2021). The abuse and overuse of antibiotics has resulted in the emergence of MDR and MAR enterococci, which has become a severe public health hazard in both humans and animals. MDR and MAR enterococci lessen the treatment option in infections developed by such strains (Obeng et al., 2013). In the present study, a high proportion of *E. faecium* isolates (80%) were phenotypically MDR in nature which reveals an alarming situation in poultry as well as humans. Previously, Tremblay et al. (2011) detected 100% MDR *E. faecium* isolates from broiler chickens in Canada. Furthermore, the index of MAR in *E. faecium* isolates was risen up to 0.83, which indicates that a high level of antibiotics was haphazardly used in the broiler chickens from where the enterococci were isolated. MDR and MAR in *E. faecium* might be developed by selective pressure triggered by the misuse, extensive use, and incorrect prescriptions of antibiotics in veterinary practices (Islam, Sobur, et al., 2021; Tawyabur et al., 2020). The MDR and MAR *E. faecium* obtained from our present study have the potential to contaminate the one‐health components. They can be transmitted to humans through the food chain or close contact with the broilers and to environments via contaminated water or feed sources.

## 5 CONCLUSION

To the best of our knowledge, we detected MDR and MAR *E. faecium* and their corresponding resistance genes for the first time in Bangladesh from healthy broiler chickens. A high level of *E. faecium* and their resistance and resistance genes detected in broiler chickens has the potential to enter into the food chain and shows a negative impact on both humans’ and animals’ health. Furthermore, the presence of MDR enterococci in broilers reveals more potential public health hazards in considering close contact with humans and animals. Though we performed our study with a limited number of broilers, this study has contributed to verify MDR enterococci and their resistance profiles in poultry. We, therefore, suggest that broiler chickens should be kept under strict biosecurity and under regular epidemiological study with a strong one‐health approach to prevent the negative effects of enterococci and to minimize the emergence of MDR and MAR *E. faecium* with their resistance genes in both humans and animals.

## CONFLICT OF INTEREST

The authors declare no conflict of interest.

## AUTHOR CONTRIBUTIONS

*Data curation, investigation, methodology, and writing‐original draft*: Krishna Roy. *Data curation, formal analysis, investigation, methodology, software, visualization, writing‐original draft, and writing‐review & editing*: Md. Saiful Islam. *Investigation*: Anamika Paul, Samina Ievy, Fatimah Muhammad Ballah, and Mithun Talukder. *Formal analysis and writing‐review & editing*: Md. Abdus Sobur. *Supervision*: Md. Shahidur Khan. *Conceptualization, formal analysis, funding acquisition, methodology, supervision, validation, writing‐original draft, and writing‐review & editing*: Md. Tanvir Rahman.

## ETHICS STATEMENT

Ethical permissions were not required during sample collection. Samples were collected with the owners’ permission.

### PEER REVIEW

The peer review history for this article is available at https://publons.com/publon/10.1002/vms3.669
